# The Association between Pretreatment anemia and Overall Survival in Advanced Non-small Cell lung Cancer: A Retrospective Cohort Study Using Propensity Score Matching

**DOI:** 10.7150/jca.55159

**Published:** 2022-01-01

**Authors:** Yucong Huang, Cuiyun Su, Huiqin Jiang, Feiwen Liu, Qitao Yu, Shaozhang Zhou

**Affiliations:** 1 Department of Respiratory Oncology, Guangxi Medical University Affiliated Tumor Hospital, No.71 Heti Road, 530021, Nanning City, Guangxi Zhuang Autonomous Region, China.; 2 Oncology Medical College, Guangxi Medical University, No.22 Shuangyong Road, 530021, Nanning City, Guangxi Zhuang Autonomous Region, China.

**Keywords:** anemia, advanced NSCLC, overall survival, prognosis, propensity score matching

## Abstract

**Background**: The purpose of this study was to investigate whether pretreatment anemia was an independent risk factor for survival in patients with advanced non-small cell lung cancer (NSCLC) after adjusting for other covariates.

**Methods**: We used propensity score matching (PSM) to minimize the influence of confounding factors and used χ2 (categorical variables), Student's t-test (normal distribution), or Mann-Whitney U test (skewed distribution) to analyze the differences among the Hb groups. Cox regression and Kaplan-Meier analyses were used to assess the association between anemia and survival. P values < 0.05 (two-sided) were considered statistically significant.

**Results:** The average age of the 758 selected participants was 58.2±11 years, and 210 patients (27.7%) had anemia. In the multivariate analysis, anemia was associated with a poor prognosis in the unmatched cohort (Hazards ratio (HR)=1.3, 95% (confidence interval (CI): 1.1-1.6; p= 0.008), and the matched cohort (HR=1.7, 95% CI: 1.3-2.3; p <0.001), emerging as an independent risk and prognostic factor in advanced NSCLC patients. In the Kaplan-Meier curve, the average survival time of anemic and non-anemic patients was 9.3 months (95% CI: 7.9-11.4 months) vs. 14.1 months (95% CI: 12-16.3 months) (p=0.0073) in the unmatched cohort. After propensity score matching, the average survival time of anemic and non-anemic patients was 10.9 months (95% CI: 8.8-12.9. months) vs. 17.8 months (95% CI: 16.0-23.3 months) (p <0.001).

**Conclusion**: Pretreatment anemia was an independent risk and prognostic factor for survival in patients with advanced NSCLC. Large-scale studies are required to confirm our findings.

## Introduction

In China, lung cancer contributes to the highest proportion of morbidity within the male population and is the second-highest cause of common malignant tumors in women 1. Furthermore, the mortality rate associated with the incidence of lung cancers ranks first in tumor-related deaths worldwide 2. Non-small cell lung cancer (NSCLC) accounts for 85% of all lung cancers, and a majority of these patients are confirmed to have an advanced stage of the disease during initial diagnosis 3. Despite rapid advancements in medical science, such as extensive use of targeted therapy and immunotherapy in clinical practice, the prognosis of advanced NSCLC remains poor, with a 5-year survival rate ranging from 5-16% 4.

Anemia is a common hematological abnormality among patients with advanced malignancies. It is reported that approximately 40-64% of the patients with malignant tumors experience anemia during the entire course of the disease 5.In a multicenter cross-sectional study conducted in China in 2012, the incidence of cancer-related anemia was 49.24% 6.

The incidence of anemia in patients with malignancies is influenced by many factors, and has not been fully clarified yet. The known causes include the tumor itself (such as blood loss, hemolysis, bone marrow invasion) and tumor treatment (such as bone marrow suppression by chemotherapy or radiotherapy, drugs that lead to impaired renal function). The deficiency of iron, folic acid, and vitamin B12 also plays an important role in the manifestation of anemia.

Several studies have shown that anemia is associated with poor overall survival (OS) in various advanced cancers, such as gastric carcinoma, prostate carcinoma, non-Hodgkin's lymphoma, and cervical cancer 7. However, the relationship between anemia and survival outcomes in patients with advanced NSCLC remains controversial. Some evidence indicates that anemia is associated with poor prognosis 8-13, while other studies failed to identify whether anemia was related to survival in patients with NSCLC 14-18. The divergence of the results in these studies may be contributed by differences in the study design, sample size, race, clinicopathological features, etc. Most importantly, the imbalance between the two groups for comparison also has a crucial influence on the results.

Thus, we performed this retrospective study with a large-scale population to evaluate whether pretreatment anemia was associated with OS in patients with advanced NSCLC, using propensity score matching (PSM) to eliminate the differences between anemia and non-anemia groups.

## Patients and Methods

### Patients

**Figure 1:**
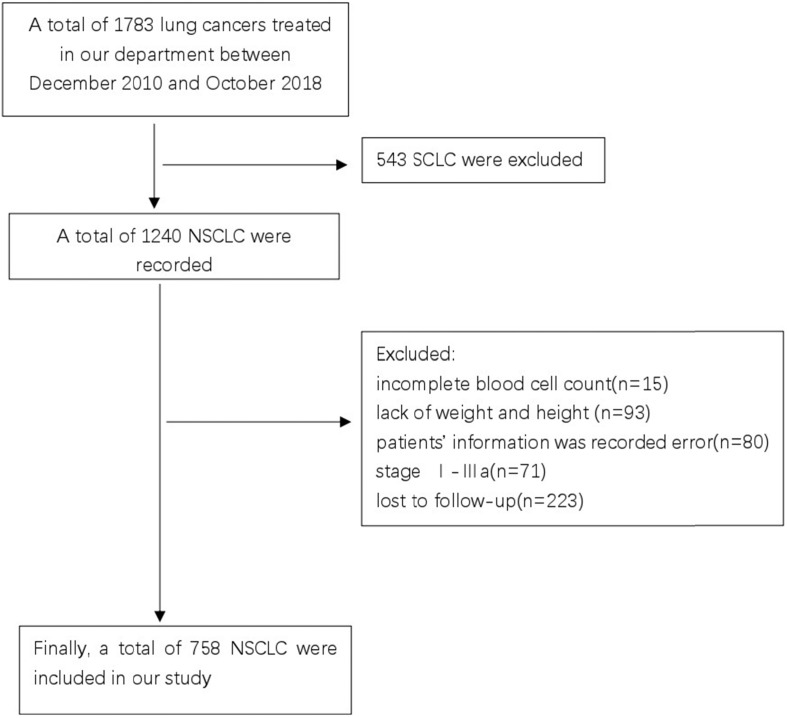
CONSORT diagram of this study.

Patients with advanced NSCLC, who were treated at the Guangxi Medical University Affiliated Cancer Hospital between December 2010 and October 2018 were screened for the study. This study was reviewed and approved by the Ethics Committee of the Hospital. Due to the retrospective nature of the cohort study, the necessity to obtain informed consent from each patient was waived. The inclusion criteria were as follows: (1) pathological or cytologically confirmed NSCLC; (2) manifestation of stage IIIB or IV NSCLC as per the seventh edition of the American Joint Committee on Cancer (AJCC) staging system; (3) availability of complete blood cell count and follow-up data; and (4) treatment-naïve, previously. The exclusion criteria were as follows: (1) complicated with hematological diseases causing anemia or renal anemia; (2) had a history of anemia before diagnosis; and (3) had no concurrent malignancy or a history of a second primary malignancy. Finally, a total of 758 patients who met the outlined criteria were enrolled in this study (Figure 1).

### Data collection and variable definitions

In our study, we summarized the following confounders that were reported in previous literature: (1) demographic characteristics; (2) variables that affect anemia or prognostic value reported in previous literature; and (3) evaluation based on our clinical experience. Therefore, the following variables were included: age, sex, smoking history, eastern cooperative oncology group performance status (ECOG PS), body mass index (BMI), pathology type, differentiation, status for anaplastic lymphoma kinase (ALK) fusion, and epidermal growth factor receptor (EGFR) mutation, sum of metastasis organs, treat type of first-line therapy, and the sum of treatment lines.

We obtained the baseline hemoglobin (Hb) levels of all subjects from the electronic medical record system of the hospital. According to the National Cancer Institute criteria and the ninth edition of expert panel in the field of Hematology in China, the definition of anemia was when Hb levels were < 12 g/dL in men and < 11 g/dL in women 19-21. In addition, OS is defined as the time from diagnosis to the date of death from any cause or the last follow-up in July 31, 2020.

The definitions of clinicopathological characteristics or parameters used in this study were based on several available classification systems. Notably, individuals who smoked more than 100 cigarettes in their lifetime were defined as smokers. Tumors were histologically classified according to the 3rd version of the World Health Organization (WHO) criteria for tumors. The performance status of patients was measured using the ECOG score. BMI was calculated by weight in kilograms divided by the square of the height in meters 22. In our study, we classified BMI into underweight (<18 kg/m2), normal weight (≤18.5-<25 kg/m2), and overweight (≥25 kg/m2) categories, respectively 23. ALK fusion was detected by fluorescence *in situ* hybridization (FISH), immunohistochemistry (IHC), or reverse transcription-polymerase chain reaction (RT-PCR) 24. Detection of EGFR sensitive mutations (19 exon deletions or 21 exon L858R) was based on the methods of amplification refractory mutation system (ARMS), droplet digital polymerase chain reaction (ddPCR), or next-generation sequencing (NGS) 25. The sum of metastasis organs was counted by organs with tumor metastasis, it should be noted that even if an organ had multiple lesions is only counted once. The first-line therapy included chemotherapy (single-drug chemotherapy or platinum-based dual-doublet chemotherapy), targeted therapy (including EGFR-TKIs or ALK-TKIs therapy), and others such as immunotherapy, radiation therapy, and antiangiogenic therapy.

Follow-up analyses were performed by the first two authors of this study. The cut-off date for the patients' follow-up was July 31, 2020. The follow-up interval was 3 months.

### Data analysis

In this study, PSM was performed to minimize the influence of confounding factors between the comparing groups, and 1:1 matching was performed between anemic and non-anemic patients, using a caliper width of 0.1 times the standard deviation of the propensity score. There were 151 matched pairs in the PSM cohort.

In the PSM cohort and adjusted cohort, we adjusted the covariates which may interfere with the results as the mixed factors, including the age, sex, smoking history, ECOG PS, BMI, pathology, differentiation, ALK fusion, EGFR mutation, sum of metastasis organs (obtained at baseline), treat type of 1st line therapy, and the sum of treatment lines.

We used χ^2^ (categorical variables) test, Student's t-test (continuous variables with normal distribution), or Mann-Whitney U test (continuous variables skewed distribution) to analyze the differences between anemia and non-anemia groups. Survival was estimated using the Kaplan-Meier method, and the difference in survival was evaluated with a stratified log-rank test. Multivariable analyses with the Cox proportional-hazards model were used to estimate the prognostic effect of anemia on survival. Covariates were included as potential confounders in the fully adjusted models to assess whether they could modify the influence of anemia on OS by more than 10% or were significantly associated with survival, with a P value less than 0.05. All analyses were performed using the statistical software packages R (http://www.R-project.org, The R Foundation) and Empower Stats (http://www.empowerstats.com, X&Y Solutions, Inc., Boston, MA). P values less than 0.05 (two-sided) were considered statistically significant.

## Results

### Clinical characteristics

In total, 758 patients with advanced NSCLC were selected for the study. The clinical characteristics are shown in Table 1. In brief, approximately 68.1% of them were men, and 210 were diagnosed with anemia at baseline (27.7%). The levels of smoking history, ECOG PS, BMI, pathology, and EGFR mutation were significantly different (p <0.05) in the pre-matched cohort, while after propensity matching, 151 pairs of patients were selected from the two groups wherein differences were reduced in all confounders and there was no statistical significance in either the anemic or the non-anemic patients.

### Univariates analysis

The results of the univariate analyses are listed in Table 2. According to the results of the univariate Cox proportional hazard model, we identified that irrespective of the matching, covariates such as smoking, ECOG PS ≥2, squamous cell carcinoma, and anemia were associated with poor prognosis in patients with advanced lung cancer. However, age ≥ 60 years (hazards ratio (HR): 1.3, 95% confidence interval (CI): 1.1-1.5; p=0.07), male sex (HR: 1.2, 95% CI: 1.0-1.5; p=0.021), sum of metastasis organs ≥ 2 (HR: 1.4, 95% CI: 1.1-1.7; p=0.008) were observed to be statistically significant before propensity score matching, while subsequent to PSM, they were not statistically significant (age ≥60 [HR: 1.3, 95% CI: 0.9-1.7; p=0.121], male sex [HR: 1.6, 95% CI: 1.1-2.2; p=0.07], and the sum of metastasis organs ≥ 2 [HR: 1.4, 95%CI: 1.0-2.1; p=0.071]). Moreover, the sum of treatment lines >3 was found to be a protective factor for survival before and after matching.

### Results of crude and adjusted Cox proportional hazards models

In our study, two models were constructed to independently analyze the effects of anemia on survival (Crude model and Adjusted model). The HRs and 95% CI are listed in Table 3. Prior to PSM, when compared with the non-anemia group, the anemia group exhibited an increased risk of death by 30% (HR:1.3, 95%CI:1.1-1.6; p=0.008). In the adjusted model before PSM, the risk of death in the anemia group was significantly higher than that in the non-anemia group (HR: 1.4, 95%CI: 1.1-1.7; p=0.013). After using PSM to minimize the covariates, the result was similar to that of the pre-match model. Anemia continued to remain a significant risk predictor for advanced NSCLC patients (HR:1.7, 95%CI:1.3-2.3; p <0.001) in the crude model and (HR:1.6,95%CI:1.2-2.2; p=0.003) in the adjusted model. Moreover, it was also found that decrease in hemoglobin levels by 1g/dL led to a concomitant increase in the risk of death by 10%.

### Subgroup analysis

**Figure 2:**
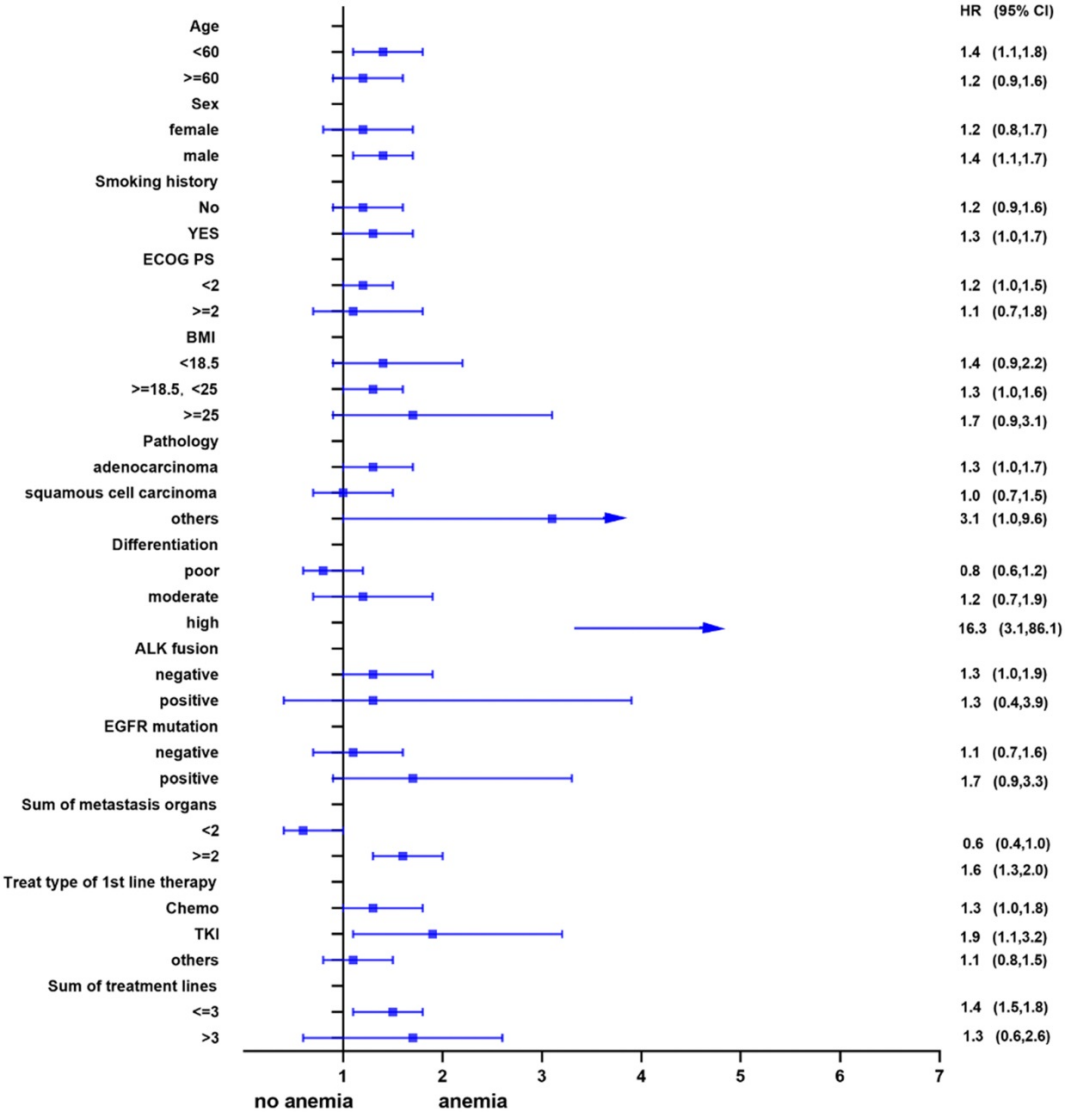
Subgroup analysis before propensity matching.

**Figure 3:**
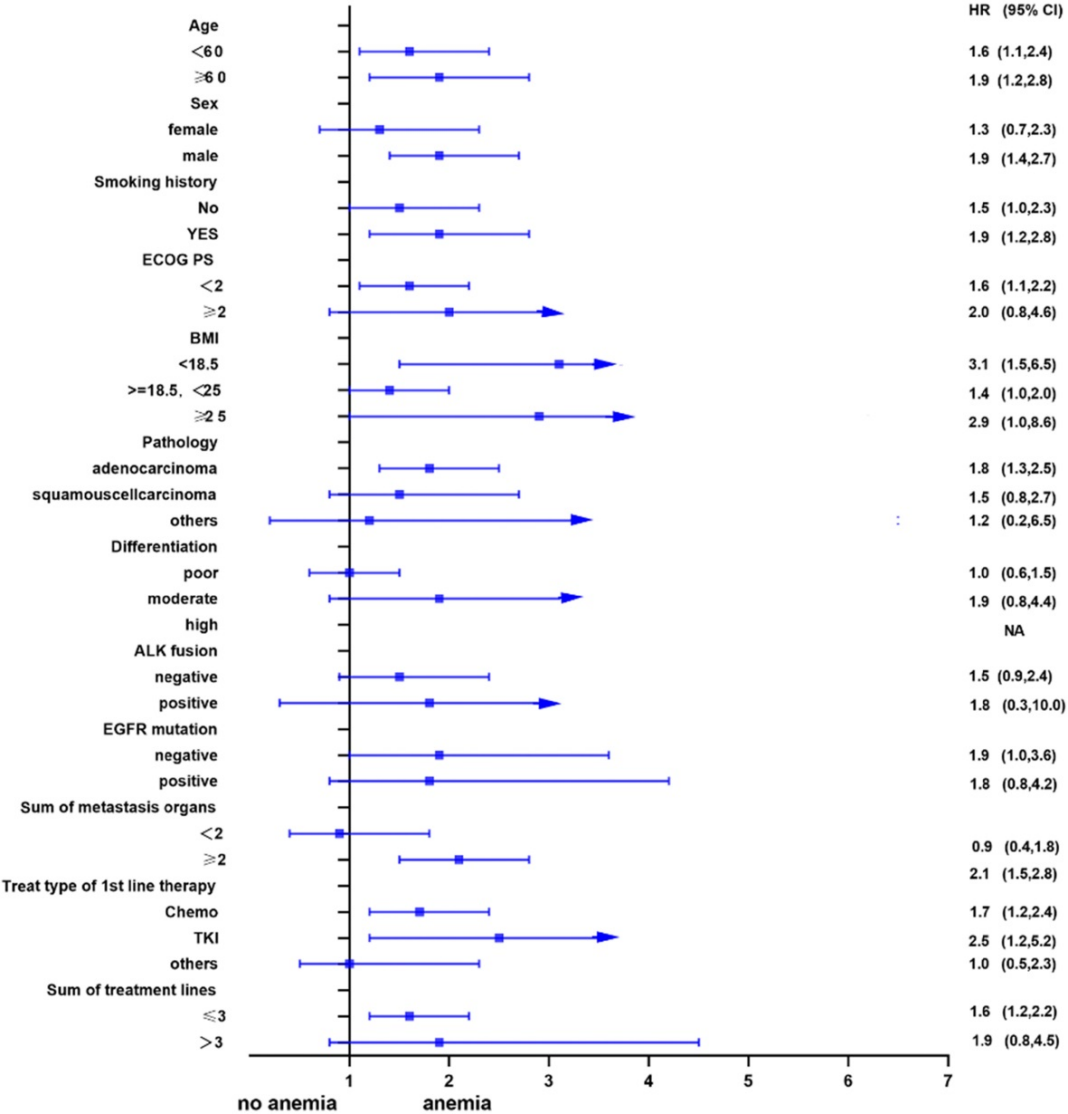
Subgroup analysis after propensity matching.

Univariate analysis showed that anemia was negatively correlated with the overall survival of the patients. Further, subgroup analyses were performed to understand this negative relationship. We used age, sex, smoking history, ECOG PS, BMI, pathology, differentiation, ALK fusion, EGFR mutation, sum of metastasis organs, treat type of 1st line therapy, and the sum of treatment lines as the stratification variables to observe the trend of effect sizes in these variables (Figure 2 and Figure 3). We noted that the majority of subgroups displayed a stable relationship between anemia and survival in the two models.

### Kaplan-Meier survival analysis

**Figure 4:**
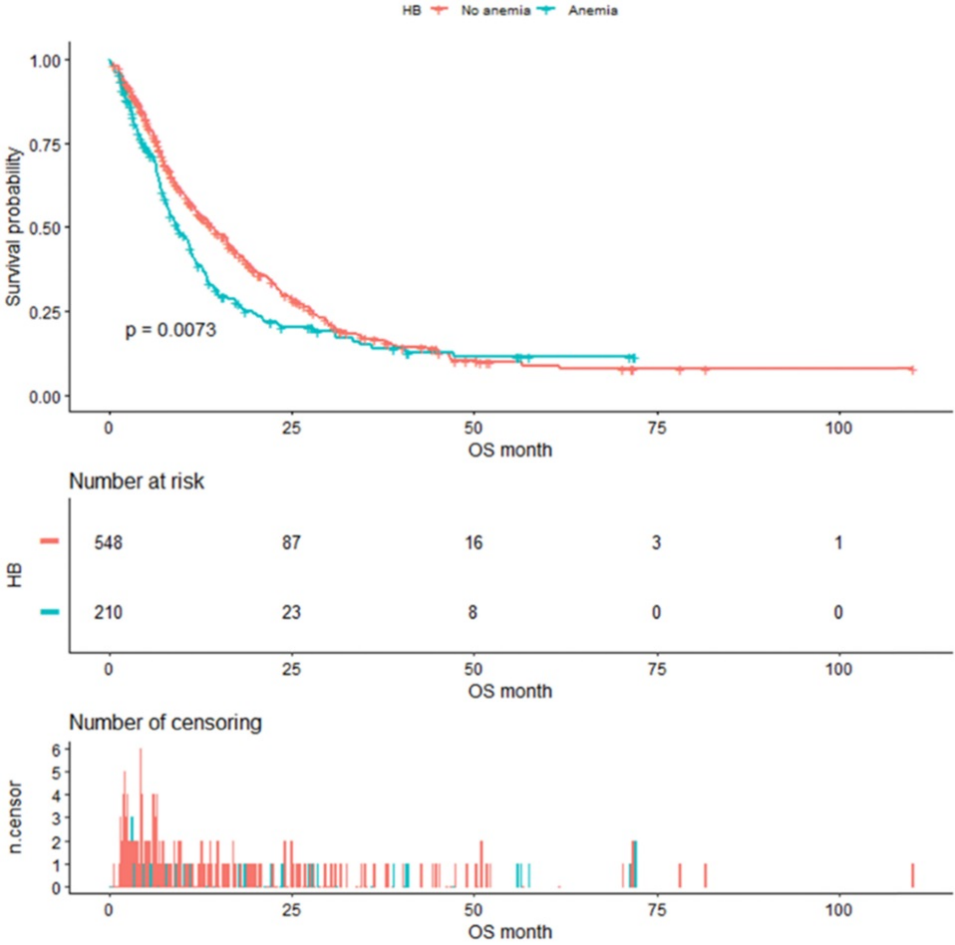
Kaplan-Meier analysis of Hb level before propensity score matching.

**Figure 5:**
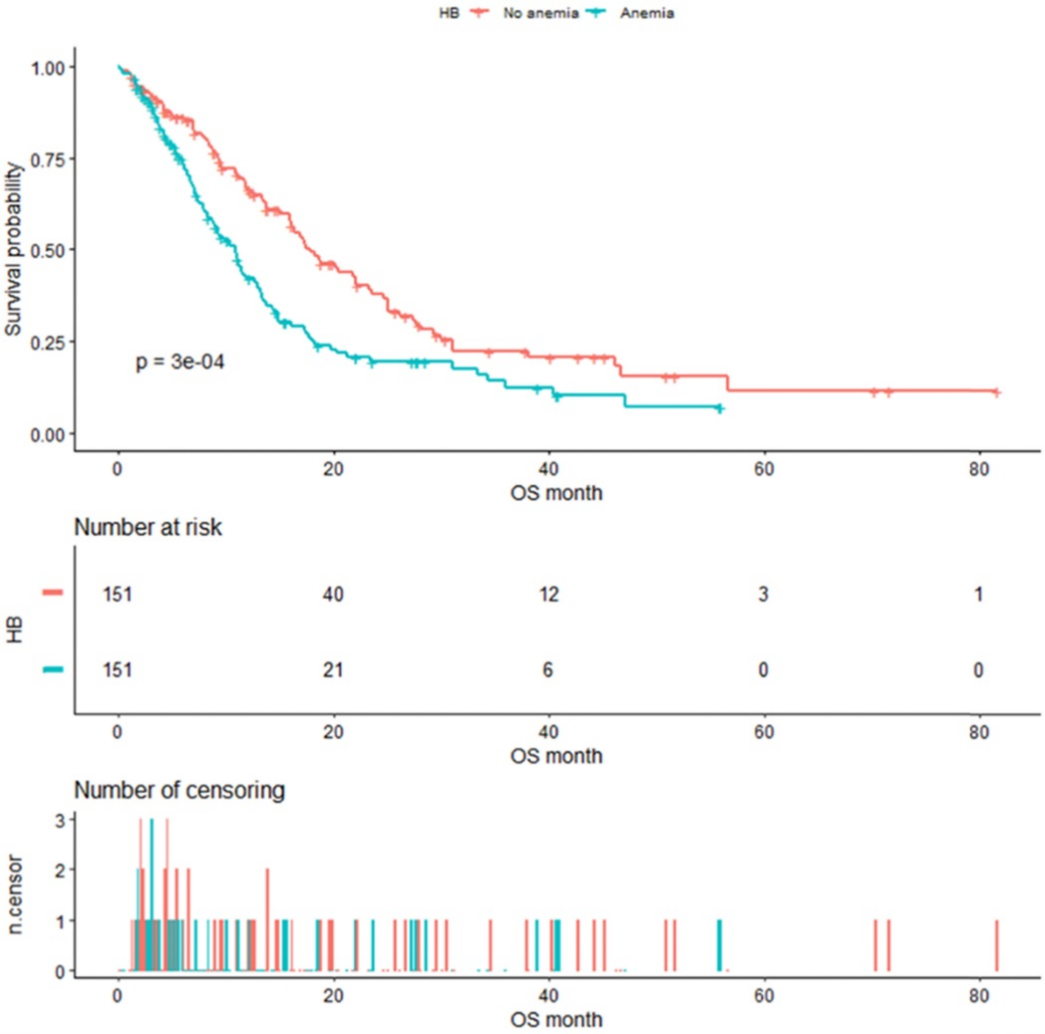
Kaplan-Meier analysis of Hb level after propensity score matching.

As shown in Figure 4, the median OS of pre-matched patients in the anemia and the non-anemia group was 9.3 months (95%CI:7.9-11.4m) and 14.1 months (95%CI:12-16.3m), respectively (P=0.0073). Notably, there was also a significant difference in the median OS between the two groups after propensity score matching (Figure 5). The median OS of patients with anemia was 10.9 months (95%CI:8.8-12.9 m) compared to 17.8 months (95%CI:16-23.3 m) in patients without anemia (P <0.001).

### Prognostic effect of Anemia on OS with different cut-off values used in other studies

Previous studies have indicated the association of anemia with shorter OS in patients with lung cancer. However, the definition of anemia varies in different studies. Therefore, as a sensitivity analysis, we used the different cut-off values proposed by those studies to identify further the prognostic effect of anemia on OS in our cohort (Table 4), and found that irrespective of the differences in definition of anemia, the results had a highly consistent HR, indicating that anemia was stably related to an unfavorable prognosis in OS.

## Discussion

Anemia, one of the most common clinical abnormalities, is often associated with the course of lung cancer. It has been reported that the prevalence of anemia in lung cancer patients is approximately 77-80% 26, 27. To determine the incidence of anemia in patients with advanced NSCLC, we studied the clinical data of patients with advanced stage IIIB - IV NSCLC, who had no previous history of anemia, and excluded the factors causing anemia by non-tumor conditions. The results showed that the prevalence of anemia among 758 patients was 27.7%. In a large-scale, prospective, and observational study conducted in Europe, the prevalence of anemia in lung cancer patients was 37.6% (753/2002) 28. In another multicenter cross-sectional study conducted in China in 2012, the incidence of anemia in lung cancer patients was even higher-50.69% (988/1949) 6. The lower percentage of anemia in our study might be attributed to our focus on hemoglobin level in pretreatment patients. Another reason might be that the patients who had a history of anemia were excluded from the study. Besides, some studies had the patients enrolled if the anemia occurred during the treatment, which may explain, in part, the differences in the occurrence rate of anemia.

The reasons for tumor-associated anemia are complex and multifactorial, such as malnutrition caused by long-term anorexia, inhibition of iron metabolism, erythropoiesis by tumor-related inflammatory factors, blood loss, and bone marrow metastasis 29, 30. Some studies suggest that the main reason for anemia is that the level of iron regulatory hormone (hepcidin) is upregulated in patients with multiple myeloma and Hodgkin's lymphoma. This can promote the transcription and synthesis of iron transporters, and eventually affect intestinal iron absorption, interfere with iron release from the monocyte-macrophage system, and disturb iron transport 29, 30. In addition, many inflammatory factors, such as interleukin-6 (IL-6), tumor necrosis factor - α, IL-1, interferon-γ, and erythropoietin (EPO) are involved in the pathogenesis of tumor-associated anemia 31, 32.

As a paraneoplastic phenomenon of tumors, anemia results in mental depression and fatigue and reduces the immunity of patients, both of which seriously affect the quality of life of patients. Meanwhile, besides contributing to tissue oxygenation disorders, anemia has been shown to aggravate tumor-associated hypoxia, stimulate tumor angiogenesis, and produce proteomic changes affecting tumor dissemination. It also affects the efficacy of radiotherapy and chemotherapy, worsening the prognosis of patients 33-35. A Japanese study revealed that the OS of patients with lung cancer and anemia was significantly shorter than patients without anemia 36. Hsu 8 et al. also identified anemia as a poor prognostic factor after conducting a study in advanced lung cancer patients aged 45 years or younger. It is worth noting that anemia was also associated with poor prognosis in NSCLC patients who received targeted therapies 13. Our study demonstrated that irrespective of the use of PSM, anemia was an independent risk factor for prognosis in patients with advanced NSCLC, as per the results of the multivariate Cox proportional hazard model. All these results indicate that anemia could enhance the aggressive behavior of the tumor and worsen physical condition of the patients.

Meanwhile, it was also observed that lower hemoglobin level was associated with worse disease prognosis in the patients. We observed that for every 1g/dL decrease in Hb, the risk of death increased by 10%. Nevertheless, Crvenkova 37 failed to demonstrate the prognostic effect of anemia in IIIA and IIIB NSCLC patients treated with chemoradiotherapy(P=0.06). Gong 16 et al. also did not find any significant correlation between anemia and survival in NSCLC patients receiving first-line chemotherapy. The divergent results of these studies may be attributed to differences in study design, scale, race, and clinicopathological features. Most importantly, the imbalance between the two groups under comparison could also have a crucial influence on the results.

To the best of our knowledge, this is the first study to employ a propensity score matching approach to analyze the relationship between pretreatment anemia and OS in patients with NSCLC. Propensity score matching was extensively used in observational studies to control confounding and minimize the differences between the two groups. To date, a growing number of studies have used propensity score matching to balance covariates across treatment groups and achieved better results. Our cohort found that using PSM to match two groups resulted in the derivation of similar conclusions.

Of note, the definition of anemia differs in different studies, which may be another critical factor influencing the final results. We used the National Cancer Institute criteria and the ninth edition of Diagnostics in China definition, which represents the Chinese population. Therefore, the corresponding findings from our observations could be helpful for decision-making in clinical practice. To eliminate the effect of different definitions of anemia on the prognosis OS, we used different cut-off values proposed by various studies as a sensitivity analysis and re-evaluated the results in our cohort. The findings showed that irrespective of the differences in definition, the results were highly consistent in HR.

Although our study adjusted for covariates against previous studies, the study still has the following limitations: (1) this was a retrospective study conducted in a single institute and did not include an independent and prospective cohort to validate the prognostic value of anemia; (2) although adjustments for potential confounders had already been carried out, there was a possibility of presence of residual or unknown confounders; (3) since the study focused on pretreatment anemia in advanced NSCLC patients, the conclusion is not suitable for patients who are in the early stage of NSCLC or present with anemia during disease after treatment; (4) data on subjects taking anti-anemic drugs during disease progression was not collected; therefore, the role of anti-anemic drugs in the improvement of clinical outcomes of the disease remains unknown and needs to be investigated further.

## Conclusion

In summary, anemia at baseline was an independent risk factor for patients with advanced NSCLC. This simple and convenient serological method of detection enabled rapid prediction of patient prognosis. In the future, extensive analyses involving a large-scale prospective cohort study are necessary to confirm our findings.

## Figures and Tables

**Table 1:** Baseline characteristics of participants.

Variables	Before propensity matching			After propensity matching		
	No anemia	Anemia	P-value	No anemia	Anemia	P-value
**Age**			0.097			0.645
<60	303(55.3%)	102(48.6%)		74(49.0%)	78 (51.7%)	
≥60	245(44.7%)	108(51.4%)		77(51.0%)	73 (48.3%)	
**Sex**			0.055			0.797
female	186(33.9%)	56 (26.7%)		43(28.5%)	41 (27.2%)	
male	362(66.1%)	154(73.3%)		108(71.5%)	110(72.8%)	
**Smoking history**			0.005			0.555
Never	284(51.8%)	85 (40.5%)		73 (48.3%)	66 (43.7%)	
Ever	259(47.3%)	119(56.7%)		76 (50.3%)	81 (53.6%)	
unknown	5 (0.9%)	6 (2.9%)		2 (1.3%)	4 (2.6%)	
**ECOG PS**			<0.001			0.171
<2	457(83.4%)	152(72.4%)		129(85.4%)	119(78.8%)	
≥2	52 (9.5%)	40 (19.0%)		11 (7.3%)	21 (13.9%)	
**unknown**	39 (7.1%)	18 (8.6%)		11 (7.3%)	11 (7.3%)	
BMI*			0.005			0.925
<18.5	67 (12.6%)	44 (22.0%)		28 (18.5%)	27 (17.9%)	
≥18.5, <25	398(74.8%)	137(68.5%)		109(72.2%)	108(71.5%)	
≥25	67 (12.6%)	19 (9.5%)		14 (9.3%)	16 (10.6%)	
**Pathology**			<0.001			0.098
adenocarcinoma	426(77.7%)	138(65.7%)		108(71.5%)	107(70.9%)	
squamous cell carcinoma	101(18.4%)	66 (31.4%)		33 (21.9%)	41 (27.2%)	
others	21 (3.8%)	6 (2.9%)		10 (6.6%)	3 (2.0%)	
**Differentiation**			0.092			0.534
poor	193(35.2%)	74 (35.2%)		47 (31.1%)	56 (37.1%)	
moderate	48 (8.8%)	31 (14.8%)		15 (9.9%)	18 (11.9%)	
high	18 (3.3%)	5 (2.4%)		5 (3.3%)	3 (2.0%)	
unknown	289(52.7%)	100(47.6%)		84(55.6%)	74(49.0%)	
**ALK fusion**			0.629			0.563
negative	213(38.9%)	74 (35.2%)		62(41.1%)	55(36.4%)	
positive	22(4.0%)	8(3.8%)		9(6.0%)	7(4.6%)	
unknown	313(57.1%)	128(61.0%)		80 (53.0%)	89 (58.9%)	
**EGFR mutation**			0.007			0.055
negative	139(25.4%)	56(26.7%)		41(27.2%)	45(29.8%)	
positive	110(20.1%)	22(10.5%)		35(23.2%)	19(12.6%)	
unknown	299(54.6%)	132(62.9%)		75(49.7%)	87(57.6%)	
**Sum of metastasis organs**			0.747			0.871
<2	102(18.6%)	43(20.5%)		27(17.9%)	29(19.2%)	
≥2	435(79.4%)	164(78.1%)		121(80.1%)	120(79.5%)	
unknown	11(2.0%)	3(1.4%)		3(2.0%)	2(1.3%)	
**Treat type of 1st line therapy**			0.556			0.891
Chemo	272(49.6%)	106(50.5%)		95(62.9%)	99(65.6%)	
TKI	90(16.4%)	28(13.3%)		28(18.5%)	26(17.2%)	
others	186(33.9%)	76(36.2%)		28(18.5%)	26(17.2%)	
Sum of treatment lines*			0.257			0.123
≤3	374(87.4%)	147(90.7%		127(84.1%)	136(90.1%)	
>3	54(12.6%)	15(9.3%)		24(15.9%)	15(9.9%)	

ECOG PS: eastern cooperative group performance status, BMI: Body mass index, EGFR: epidermal growth factor receptor, ALK: Anaplastic Lymphoma Kinase, Treatment type of first-line therapy: Chemotherapy, Targeted therapy, others). BMI* and Sum of treatment lines* had missing data. *p values<0.05 were considered statistically significant.

**Table 2:** Univariate and analysis of overall survival.

Variables	Before propensity matching			After propensity matching		
	Statistics	HR(95%CI)	P-value	Statistics	HR(95%CI)	P-value
**Age**						
<60	405 (53.4%)	1		152 (50.3%)	1	
≥60	353 (46.6%)	1.3 (1.1, 1.5)	0.007	150 (49.7%)	1.3 (0.9, 1.7)	0.121
**Sex**						
female	242 (31.9%)	1		84 (27.8%)	1	
male	516 (68.1%)	1.2 (1.0, 1.5)	0.021	218 (72.2%)	1.6 (1.1, 2.2)	0.07
**Smoking history**						
Never	369 (48.7%)	1		139 (46.0%)	1	
Ever	378 (49.9%)	1.4 (1.2, 1.7)	<0.001	157 (52.0%)	1.7 (1.3, 2.3)	<0.001
unknown	11 (1.5%)	4.0 (2.2, 7.3)	<0.001	6 (2.0%)	4.4 (1.9, 10.2)	<0.001
**ECOG PS**						
<2	609 (80.3%)	1		248 (82.1%)	1	
≥2	92 (12.1%)	1.5 (1.1, 1.9)	0.003	32 (10.6%)	1.9 (1.2, 2.9)	0.003
unknown	57 (7.5%)	1.3 (1.0, 1.8)	0.051	22 (7.3%)	1.4 (0.9, 2.3)	0.151
BMI*						
<18.5	111 (15.2%)	1		55 (18.2%)	1	
≥18.5, <25	535 (73.1%)	0.9 (0.7, 1.1)	0.351	217 (71.9%)	0.9 (0.6, 1.3)	0.688
≥25	86 (11.7%)	0.7 (0.5, 1.0)	0.049	30 (9.9%)	0.6 (0.4, 1.1)	0.127
**Pathology**						
adenocarcinoma	564 (74.4%)	1		215 (71.2%)	1	
squamous cell carcinoma	167 (22.0%)	1.4 (1.2, 1.8)	0.001	74 (24.5%)	1.6 (1.1, 2.2)	0.006
others	27 (3.6%)	1.2 (0.7, 1.8)	0.512	13 (4.3%)	1.4 (0.7, 2.7)	0.311
**Differentiation**						
poor	267 (35.2%)	1		103 (34.1%)	1	
moderate	79 (10.4%)	0.8 (0.6, 1.1)	0.22	33 (10.9%)	0.8 (0.5, 1.2)	0.292
high	23 (3.0%)	0.8 (0.5, 1.3)	0.449	8 (2.6%)	1.2 (0.6, 2.6)	0.64
unknown	389 (51.3%)	0.7 (0.6, 0.8)	<0.001	158 (52.3%)	0.7 (0.5, 1.0)	0.059
**ALK fusion**						
negative	287 (37.9%)	1		117 (38.7%)	1	
positive	30 (4.0%)	0.7 (0.4, 1.2)	0.154	16 (5.3%)	0.5 (0.2, 1.3)	0.155
unknown	441 (58.2%)	1.2 (1.0, 1.5)	0.046	169 (56.0%)	1.4 (1.0, 1.9)	0.026
**EGFR mutation**						
negative	195 (25.7%)	1		86 (28.5%)	1	
positive	132 (17.4%)	0.6 (0.4, 0.8)	<0.001	54 (17.9%)	0.7 (0.5, 1.2)	0.219
unknown	431 (56.9%)	1.2 (1.0, 1.5)	0.093	162 (53.6%)	1.3 (0.9, 1.8)	0.127
**Sum of metastasis organs**						
<2	145 (19.1%)	1		56 (18.5%)	1	
≥2	599 (79.0%)	1.4 (1.1, 1.7)	0.008	241 (79.8%)	1.4 (1.0, 2.1)	0.071
unknown	14 (1.8%)	1.3 (0.7, 2.4)	0.348	5 (1.7%)	1.9 (0.7, 5.3)	0.234
**Treat type of 1st line therapy**						
Chemo	378 (49.9%)	1		194 (64.2%)	1	
TKI	118 (15.6%)	1.0 (0.7, 1.2)	0.754	54 (17.9%)	1.0 (0.7, 1.4)	0.916
others	262 (34.6%)	1.4 (1.1, 1.7)	0.001	54 (17.9%)	1.0 (0.6, 1.5)	0.911
Sum of treatment line*						
≤3	521 (88.3%)	1		263 (87.1%)	1	
>3	69 (11.7%)	0.5 (0.4, 0.7)	<0.001	39 (12.9%)	0.4 (0.3, 0.6)	<0.001
**Hb**						
No anemia	548 (72.3%)	1		151 (50.0%)	1	
Anemia	210 (27.7%)	1.3 (1.1, 1.6)	0.008	151 (50.0%)	1.7 (1.3, 2.3)	<0.001

ECOG PS: eastern cooperative group performance status, BMI: Body mass index, EGFR: epidermal growth factor receptor, ALK: Anaplastic Lymphoma Kinase, Treatment type of first-line therapy: Chemotherapy, Targeted therapy, others). BMI* and Sum of treatment lines* had missing data *p values<0.05 were considered statistically significant.

**Table 3:** Effect of Anemia on OS in entire and matched cohorts: results of Cox proportional hazard model.

Variables	Before propensity matching				After propensity matching			
	Crude model		Adjusted model		Crude model		Adjusted model	
	HR(95%CI)	P-value	HR(95%CI)	P-value	HR(95%CI)	P-value	HR(95%CI)	P-value
No anemia	1		1		1		1	
Anemia	1.3(1.1,1.6)	0.008	1.4(1.1,1.7)	0.013	1.7(1.3, 2.3)	<0.001	1.6(1.1,2.2)	0.003
Hb(per 1g/dl decrease)	1.1(1.0,1.1)	<0.001	1.1(1.1,1.2)	<0.001	1.1(1.0, 1.2)	0.001	1.1(1.0,1.2)	0.018

Crude mode adjusted for: None. Adjusted model adjusted for: Age; Sex; Smoking history; ECOG PS; BMI; Pathology; Differentiation; ALK fusion; EGFR mutation; Sum of metastasis organs; Treat type of 1st line therapy; Sum of treatment lines *p values<0.05 were considered statistically significant.

**Table 4:** Effect of Anemia on overall survival with using other studies' cut-off: Cox proportional hazard modeling results.

First author	No of cases	Anemia Cut Off	Outcome	Other Studies		Ours	
				HR(95%CI)	p-value	HR(95%CI)	p-value
Holgersson et al^38^	835	<11g/dL	OS	1.33(1.10,1.61)	0.003	1.3 (1.0, 1.7)	0.059
Pathak et al^9^	752	<12 g/dL	OS	1.51(1.03,2.21)	0.030	1.4 (1.1, 1.8)	0.002
Belbaraka et al^11^	45	male<13g/dL,	OS	2.78(1.29,5.99)	0.009	1.4 (1.1, 1.9)	0.009
		female<11.5g/dL.					
Park et al^13^	630	male<13g/dL,	OS	1.31(1.03,1.68)	0.030	1.4 (1.1, 1.7)	0.004
		female<12g/dL.					

Our cohort were adjusted for: Age; Sex; Smoking history; ECOG PS; BMI; Pathology; Differentiation; ALK fusion; EGFR mutation; Sum of metastasis organs; Treat type of 1st line therapy; Sum of treatment lines.

## Abbreviations

NSCLC: Non-small cell lung cancer
PSM: Propensity score matching
HR: Hazards ratio
CI: Confidence interval
OS: Overall survival
AJCC: American Joint Committee on Cancer
ECOG PS: Eastern cooperative oncology group performance status
BMI: Body mass index
ALK: Anaplastic lymphoma kinase
EGFR: Epidermal growth factor receptor
HB: Hemoglobin
WHO: World Health Organization
FISH: Fluorescence *in situ* hybridizations
IHC: Immunohistochemistry
RT-PCR: Reverse transcription-polymerase chain reaction
ARMS: Amplification refractory mutation system
ddPCR: Droplet digital polymerase chain reaction
NGS: Next-generation sequencing
EPO: Erythropoietin
